# Serum Cystatin C as an Early Predictive Biomarker for Acute Kidney Injury (AKI) in Neonates: A PRISMA-DTA Systematic Review

**DOI:** 10.3390/children13091202

**Published:** 2026-09-05

**Authors:** Diana Cristina Potîrcǎ, Ioana Andrada Radu, Dumitru Alin Teacoe, Rareș Arseniu, Radu Galiș, Boris W. Kramer, Maria Livia Ognean

**Affiliations:** 1 Doctoral School of Medicine, Faculty of Medicine, Lucian Blaga University of Sibiu, 550024 Sibiu, Romania; dianacristina.potirca@ulbsibiu.ro (D.C.P.); dumitrualin.teacoe@ulbsibiu.ro (D.A.T.); rares.arseniu@ulbsibiu.ro (R.A.); 2 Faculty of Medicine, Lucian Blaga University of Sibiu, 550024 Sibiu, Romania; maria.ognean@ulbsibiu.ro; 3 Department of Neonatology, Clinical County Emergency Hospital, 550245 Sibiu, Romania; 4 Department of Medical Sciences, Faculty of Medicine, Oradea University, 410087 Oradea, Romania; 5 Department of Neonatology, Emergency County Hospital Bihor, 410167 Oradea, Romania; 6 Department of Neonatology, Poznan University of Medical Sciences, 61-701 Poznan, Poland; bkramer@pums.edu.pl

**Keywords:** acute kidney injury, serum cystatin C, diagnostic accuracy, neonate, systematic review

## Abstract

**Highlights:**

**What are the main findings?**

- Previous reviews of serum cystatin C (sCysC) in neonatal acute kidney injury (AKI) pooled studies with heterogeneous sampling timing designs, without distinguishing early predictive performance from concurrent diagnostic values.
- sCysC showed good diagnostic accuracy for concurrent AKI detection and, in several high-risk populations, was elevated at earlier scheduled sampling timepoints than serum creatinine.

**What are the implications of the main findings?**

- Standardized assay methods and validated, population-specific thresholds are necessary before sCysC can be routinely used to detect neonatal AKI.
- Prospective multicenter studies are required to assess whether sCysC-guided monitoring improves neonatal outcomes.

**Abstract:**

Background: Acute kidney injury (AKI) affects 12–40% of critically ill newborns and significantly increases mortality risk. Diagnosis currently relies on serum creatinine (sCr), which rises only after substantial renal damage, so early biomarkers are needed in this vulnerable population. Serum cystatin C (sCysC) is a candidate because it is produced at a constant rate by all nucleated cells. Aim: The study aimed to investigate the diagnostic performance of sCysC for early detection of neonatal AKI. Methods: This systematic review followed the PRISMA-DTA guidelines and is registered with PROSPERO (CRD420261302574). We searched four databases for studies measuring sCysC in neonates (0–28 days) with validated assays and creatinine-based AKI definitions as the reference standard. Studies were categorized by sampling timing as concurrent diagnostic or early predictive, and appraised with QUADAS-2 and QUIPS, respectively. Results: Thirteen studies were included. In the early predictive group, sCysC was elevated at scheduled timepoints preceding creatinine-defined AKI, with AUC values of 0.670–1.000. The concurrent diagnostic studies reported sensitivities of 84.8–88.5%, specificities of 61.8–75.0%, and AUC values of 0.844–0.849. Reported cut-offs ranged from 0.60 to 2.87 mg/L, with one sepsis-associated AKI study reporting 9.4 mg/L. Conclusions: sCysC is a promising adjunctive biomarker whose earlier elevation may support earlier recognition of neonatal AKI. Standardized assays and validated population-specific thresholds are required before routine clinical use.

## 1. Introduction

Acute kidney injury is an important complication among the neonatal population inside the neonatal intensive care unit (NICU). It is a significant cause of morbidity and mortality; therefore, early detection is essential for timely intervention [1,2,3]. Nowadays, the assessment of AKI in neonates is challenging since the diagnosis relies primarily on traditional biomarkers, which include serum creatinine (sCr) levels, and urine output (oliguria/anuria) [4,5]. Currently, neonatal AKI is classified using standardized systems such as the neonatal-modified KDIGO (Kidney Disease: Improving Global Outcomes), AKIN (Acute Kidney Injury Network), and pRIFLE (pediatric Risk, Injury, Failure, Loss, End-Stage Renal Disease) [6,7]. These systems define and stage AKI severity based on the magnitude of sCr increase from baseline and/or the duration of diminished urine output [6,8,9].

However, the traditional biomarkers are limited by multiple disadvantages such as the following:

- Maternal influence: in the first 24 to 72 h of life, neonatal sCr represents maternal renal function and not the infant’s, as creatinine freely crosses the placenta;
- Gestational age dependence: sCr levels decline and stabilize at varying rates over the first few weeks of life, meaning its baseline value is highly affected by gestational age (GA);
- Delayed detection: sCr is a late AKI marker as it measures function rather than actual tissue damage; sCr often does not rise until 48 to 72 h after the renal injury, typically its levels rise only after almost half of the nephrons have already been compromised;
- Non-renal factors: sCr production and concentration depend on variables such as muscle mass, hydration status, age, and gender [10,11,12,13].

Recent research has focused on serum cystatin C (sCysC) as a potential early biomarker for AKI in neonates. Serum cystatin C is a 13.3 kDa cationic protein produced at a constant rate by all nucleated cells. Due to its small size, sCysC is freely filtered through the glomerular membrane and is subsequently completely reabsorbed and catabolized by the proximal renal tubules, without undergoing tubular secretion [13].

Serum cystatin C has the following advantages over other biomarkers: it is minimally affected by muscle mass and maternal renal function, it is not significantly influenced by gender, gestational age, muscle mass, and hydration status, and reacts rapidly to alterations in the glomerular filtration rate (GFR) [4,13,14]. In order to achieve an early diagnosis, the use of sCysC instead of sCr in the neonatal population presents fundamental physiological advantages.

Despite no current consensus, multiple reviews and primary clinical studies suggest that sCysC may be an effective early biomarker for AKI in neonates, owing to its higher sensitivity than traditional biomarkers [6,15,16]. There is a growing interest in sCysC as a biomarker for neonatal AKI; however, essential aspects remain insufficiently clarified. The most important ones are when, how, and in which neonatal populations sCysC should be used. There are currently no standard threshold values for sCysC to accurately diagnose neonatal AKI. Another problem is the fact that most of the studies were conducted on a mixed population, both full-term and preterm neonates, who have different degrees of renal maturation [16] AKI diagnosis is usually established using modified neonatal KDIGO, AKIN, or pRIFLE criteria, but the reference standards (including measurement time points) vary considerably across studies [1].

Previous reviews pooled data from studies with heterogeneous time points of sCysC measurements [13]. Among those reviews there was no distinction between the biomarker’s capacity to predict AKI before clinical manifestation and its ability to confirm AKI concurrently diagnosed with creatinine-based criteria. As the clinical implications of these two biomarkers are fundamentally different, this represents a gap in the literature.

Consequently, an important question remains unanswered: does sCysC perform better as an early predictor of neonatal AKI or as a concurrent diagnostic marker?

This review investigated the effectiveness and the role of sCysC in early AKI detection in newborns. We attained a structured narrative synthesis by systematically analyzing the existing studies and separating early predictive performance from concurrent diagnostic values of this biomarker.

## 2. Methods

A. Protocol and Registration:

This systematic review was conducted and reported in accordance with the Preferred Reporting Items for Systematic Reviews and Meta-Analyses for Diagnostic Test Accuracy (PRISMA-DTA) guidelines [17]. The completed PRISMA-DTA checklist is provided in Supplementary Material File S1. The narrative synthesis adhered to the Synthesis Without Meta-analysis (SWiM) reporting guideline [18] (Supplementary Material File S2). The protocol was prospectively registered in the PROSPERO International Prospective Register of Systematic Reviews (Registration ID: CRD420261302574).

B. Eligibility criteria

Studies were eligible for this systematic review if they met the following criteria:

(a) Population: Neonates from birth to 28 days of postnatal age. Both term (≥37 weeks) and preterm (<37 weeks) neonates across all clinical settings were considered (e.g., NICU, cardiac surgery). A minimum sample size of 10 neonates was required. This threshold was settled to minimize the risk of small studies’ effects. Exclusion criteria involved mixed pediatric populations without extractable neonatal data; primary congenital kidney anomalies; studies focused exclusively on chronic kidney disease.
(b) Index test: Serum cystatin C (sCysC) measured by any validated method (e.g., PENIA, PETIA, ELISA). Given the potential validation in calibration between different assays, the specific measurement method was extracted from each study and treated as a potential source of methodological heterogeneity. Studies assessing only urinary cystatin C or reporting only cystatin C-based estimated glomerular filtration rate (eGFR) without extractable data for diagnostic accuracy were also excluded.
(c) Reference standard: The reference standard used for AKI diagnosis was defined by validated sCr and/or urine output criteria, including the neonatal-modified KDIGO, original KDIGO, AKIN, RIFLE, or pRIFLE criteria. Baseline sCr was required to be defined using postnatal measurements obtained after the initial period of maternal creatinine influence (typically after 24–48 h of life).
(d) Target condition: Neonatal AKI.
(e) Study design: Prospective and retrospective cohort studies, cross-sectional diagnostic accuracy studies reporting sensitivity, specificity, AUC-ROC (area under the receiver operating characteristic curve), cut-off value.
(f) Outcomes: Diagnostic accuracy measured through various metrics including sensitivity, specificity, AUC-ROC, and optimal threshold values.

C. Information sources and search strategy:

(a) Information Sources Databases: A comprehensive systematic search was conducted in the following electronic databases: PubMed/MEDLINE (1.02.2026), Cochrane Central Register of Controlled Trials/CENTRAL (2.02.2026), Web of Science (2.02.2026), and Scopus (8.03.2026).
(b) Search strategy: An extensive literature search was conducted to identify studies evaluating the predictive and diagnostic value of sCysC for AKI in neonates. The search strategy was developed using three core concepts combined with Boolean operators: (1) neonatal population terms, (2) sCysC biomarker terms, and (3) AKI. Data-specific syntax and controlled vocabulary were applied for each involved database, including Medical Subject Headings (MeSH).
  Complete search strategies for all databases are provided in Supplementary Material File S3.
(c) Limits and Restrictions: During the initial search, no language or publication date restrictions were applied. No study design filters were applied.

D. Study Selection and Data Extraction:

All records retrieved from the database searches were imported into Zotero for de-duplication. Study selection was conducted in two sequential stages: (1) title and abstract screening, followed by (2) full-text eligibility assessment.

The data extraction after the screening was completed using a standardized extraction form, which was initially tested on a subset of studies to ensure consistency. Extracted data included study characteristics, population demographics, assay methods for sCysC, AKI definitions, timing of biomarker measurement, and diagnostic accuracy outcomes, including true positives, false positives, false negatives, true negatives, sensitivity, specificity, and AUC-ROC.

Both screening stages and data extraction were performed independently by two reviewers using Rayyan, with reviewers blinded to each other’s decisions. Disagreements were resolved through discussion with a third reviewer.

E. Relationship between index test and reference standard according to timing:

The selected studies were grouped into two categories based on the timing between sCysC measurement (index test) and AKI diagnosis using sCr (reference standard):

- Concurrent diagnostic accuracy studies: sCysC and sCr were measured simultaneously or within 24 h, enabling direct comparison of the biomarkers. This design assessed the diagnostic accuracy of sCysC in detecting AKI, concurrently with the reference standard.
- Predictive accuracy studies: sCysC was measured more than 24 h before AKI was diagnosed using creatinine-based criteria. This design evaluated the ability of sCysC to identify newborns at risk of developing AKI before traditional biomarkers had abnormal levels.

This time-related classification highlighted the dual clinical utility of sCysC: earlier measurement assessed its value as a predictive biomarker, while concurrent measurement assessed its value as a diagnostic method.

The 24 h window is a classification threshold adopted for this review rather than an empirically derived cut-point. It was informed by studies reporting sCysC elevation one to two days before serum creatinine in neonates, including El-Gammacy et al. [10] and Refat et al. [2]. Both sampled at pre-specified timepoints rather than tracking paired timing within individual neonates, so the intervals they report describe a sampling schedule and not a measured lead interval. The threshold therefore separates measurements obtained before the reference-standard diagnosis from those obtained alongside it, and carries no claim about how much earlier an individual neonate crosses a diagnostic threshold.

F. Risk-of-Bias Assessment

The risk-of-bias of the included studies was evaluated by two independent assessors using the Quality Assessment of Diagnostic Accuracy Studies 2 (QUADAS-2) tool for the concurrent diagnostic accuracy studies and the Quality In Prognosis Studies (QUIPS) tool [19] for the early predictive studies, with signaling questions adapted to the neonatal AKI topic. Disagreements were resolved through discussion and consensus, or through discussion with a third reviewer. Studies employing case–control designs that included completely healthy neonates as controls were flagged as having potential spectrum bias. Studies were additionally evaluated for potential treatment-informed bias, defined as situations where sCysC results were available to clinicians and may have influenced clinical management, potentially affecting subsequent creatinine-based AKI diagnosis.

G. Data synthesis and analysis

The previous literature demonstrated significant heterogeneity, which prevented meta-analysis. This systematic review also identified substantial clinical and methodological heterogeneity among included studies. Key sources included variability in AKI definitions, differences in sCysC assessment and cut-off values, diverse study populations, and inconsistent timing between index test measurement and reference standard assessment. Heterogeneity was described narratively rather than pooled statistically. Reported diagnostic performance varied considerably; in the predictive group, AUC values ranged from 0.670 to 1.00, reflecting differences in population, clinical context, AKI definition, and measurement timing that could not be addressed through statistical pooling. Data were synthesized using a structured narrative synthesis approach in accordance with the Synthesis Without Meta-analysis (SWiM) reporting guideline. To address the primary research question and to preserve the distinction between clinical utilities, the studies evaluating concurrent diagnostic accuracy and those who assessed early predictive accuracy were analyzed separately.

Diagnostic performance metrics were summarized and they included sensitivity, specificity, positive and negative predictive values, and area under the receiver operating characteristic curve (AUC).

Sensitivity and Subgroup Analyses

To explore heterogeneity and assess the robustness of findings, the following narrative sensitivity analyses had been pre-specified: (1) exclusion of case–control studies to evaluate the impact of spectrum bias on diagnostic accuracy estimates; (2) stratification by sCysC assay method (e.g PENIA, ELISA, PETIA) to account for calibration variances; and (3) exclusion of studies with small sample size (n < 30) to assess the influence of imprecise estimates on overall findings. A predefined subgroup analysis was established to evaluate the diagnostic accuracy of sCysC separately for preterm and term neonates, because of the different degrees of renal maturation.

There were no deviations from the prospectively registered PROSPERO protocol occurring during the conduct of this systematic review.

## 3. Results

### 3.1. Study Selection

**Figure 1:**
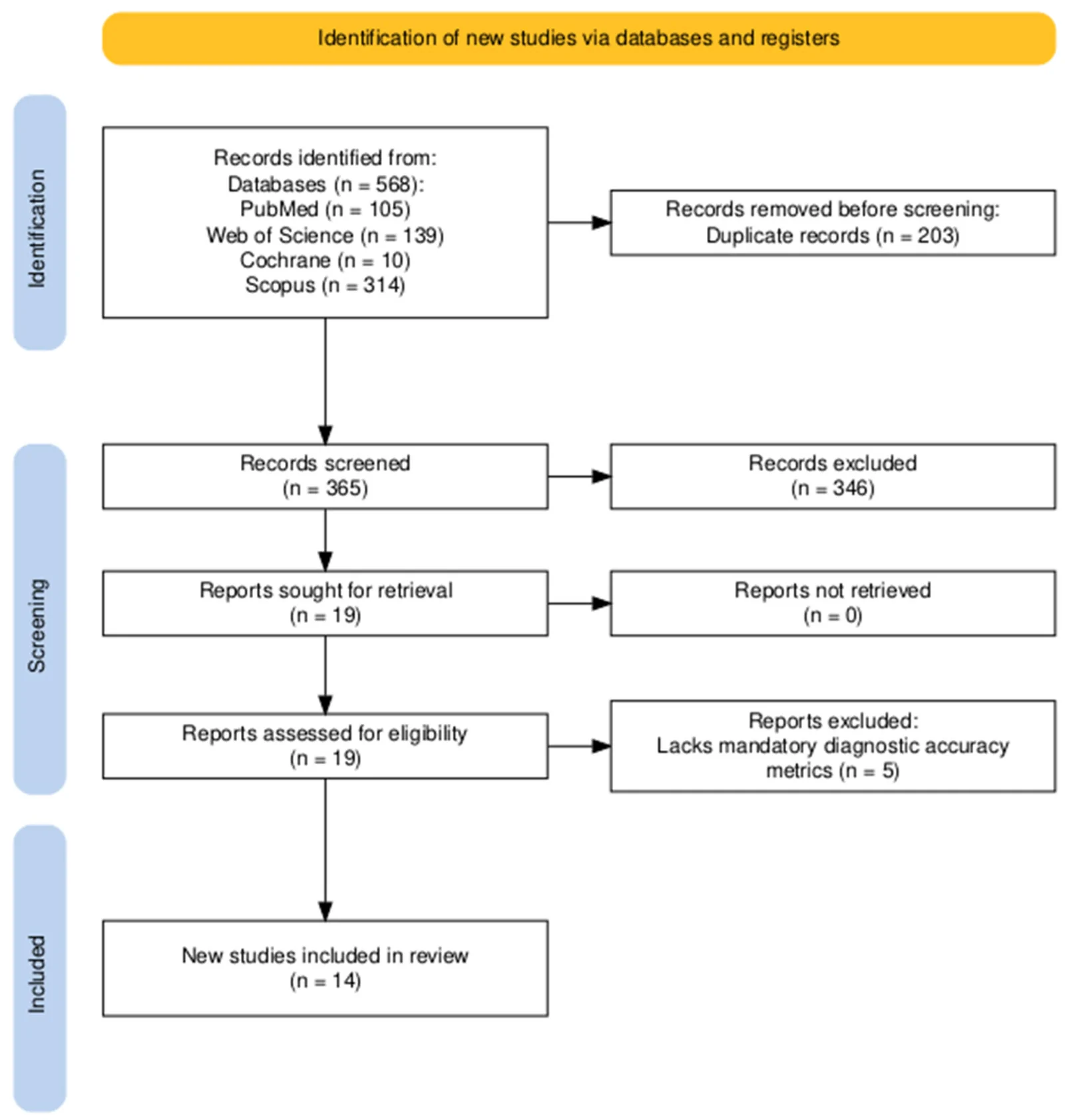
PRISMA-DTA flow diagram of study identification, screening, eligibility, and inclusion.

We conducted a systematic literature search using the selected electronic databases and identified a total of 568 records (PubMed 105 records, Web of Science 139 records, Cochrane CENTRAL 10 records, and Scopus 314 records) (Figure 1). After removal of 203 duplicate records, 365 records remained for title and abstract screening.

During the initial screening phase, 346 records were excluded based on the eligibility criteria: mixed pediatric population, absence of sCysC measurement, and non-eligible designs (e.g., reviews, meta-analyses, editorials, or animal studies). We therefore assessed the full text of the remaining 19 articles. Six reports were excluded at this stage. Four lacked extractable diagnostic accuracy metrics. Two were excluded on design: Xu et al. [13] proposes a cystatin C-based AKI definition evaluated against mortality outcomes rather than testing sCysC against a creatinine-based reference standard, and Treiber et al. [20] reports receiver operating characteristic analysis discriminating asphyxiated from healthy neonates rather than neonates with from neonates without AKI, so it provides no accuracy estimate against a creatinine-based reference standard. The reasons for each exclusion are detailed in Supplementary Material Table S1.

Thirteen studies met all inclusion criteria and were included in the final synthesis. The study selection process is illustrated in the PRISMA-DTA flow diagram (Figure 1).

### 3.2. Characteristics of the Included Studies

The 13 included studies were published between 2012 and 2025, showing a rising interest in new AKI biomarkers for the neonatal population. Studies were conducted in five countries: Egypt (n = 8), China (n = 2), Turkey (n = 1), Greece (n = 1), and Indonesia (n = 1). Seven studies used prospective case–control designs; the remainder were prospective or observational cohort-type and cross-sectional diagnostic accuracy studies. Enrolment ranged from 30 to 200 neonates, totaling 1253. Because several studies enrolled healthy controls alongside the clinical population, the denominators for the accuracy analyses are smaller than the enrolment figures and are given per study in Supplementary Material Table S2.

Population. Relating to gestational age, seven studies enrolled only term neonates, four studies included only preterm neonates, and two studies included mixed populations. The clinical context varied: perinatal asphyxia or HIE (n = 4), RDS (n = 4), critically ill neonates (n = 2), sepsis-associated AKI (n = 1), severe hyperbilirubinemia (n = 1), and a mixed NICU population (n = 1).

Index test. The methods used for measuring sCysC were as follows enzyme-linked immunosorbent assay (ELISA) in eight studies, particle-enhanced nephelometric immunoassay (PENIA) in three studies (including one fully automated N Latex assay), particle-enhanced turbidimetric or latex-enhanced immunoturbidimetric methods in two studies. Sampling timing varied widely and is given per study in Table 1. The earliest measurements were obtained within the first 24 h of life or of admission (Refat et al. [2], Zhang et al. [21], Wang et al. [22], Abdelsattar et al. [15], and the first timepoint of Nour et al. [23]); most studies sampled at least once within the first three days; and two extended beyond the first week, Elmas et al. [24] sampling again at day 30 and Hidayati et al. [25] sampling across the first 28 days of life. Reported threshold values ranged from 0.60 to 2.87 mg/L, with one outlying threshold of 9.40 mg/L.

Reference standard. KDIGO or KDIGO-adapted neonatal criteria were used in six studies to define AKI, pRIFLE/nRIFLE/AKIN criteria in four studies, and institution-specific definitions or absolute sCr thresholds in three studies (Elmas et al. [24], oliguria and/or a rise in sCr; Sarafidis et al. [26]; El-Frargy et al. [27]). Among the cohort-type studies, the proportion of neonates developing AKI ranged from 17.3% (El-Gammacy et al. [10]) to 52% (Abdelsattar et al. [15]); in the case–control designs, this proportion is fixed by sampling and does not estimate incidence.

Classification based on sampling timing. Based on the timing between sCysC measurement and AKI diagnosis, 3 studies evaluated concurrent diagnostic accuracy (sCysC measured within the same 24 h window as AKI diagnosis) and 11 evaluated early predictive accuracy (sCysC measured more than 24 h before AKI diagnosis), with one study [15] contributing to both analyses because of its mixed timing design.

The detailed characteristics of all included studies are summarized in Table 1.

**Table 1:** Characteristics of included studies.

Study	Country	Design	Population	Clinical Context	sCysC Assay Method	Timing of sCysC Measurement	Cut-Off (mg/L)	AKI Reference Standard
Abdelaal et al., 2017 [16]	Egypt	Prospective case–control	Preterm (100)	RDS	N latex sCysC test kit and a fully automated particle enhanced nephelometric immunoassay	DOL 1, 3, 7	DOL-3 ≥1.28	KDIGO
El-Gammacy et al., 2018 [10]	Egypt	Prospective	Preterm (75)	RDS	ELISA	DOL 3	1.3	Modified (pRIFLE)
El-Sadek et al., 2020 [27]	Egypt	Multicenter, prospective, case–control	Term (90)	critically ill	ELISA	Day 1, 3, 5 of admission	2.68	KDIGO
Elmas et al., 2013 [24]	Turkey	Prospective case–control	Preterm (62)	RDS	PENIA	DOL 3, 30	1.62	Oliguria and/or rise in sCr
Nour et al., 2020 [23]	Egypt	Prospective observational	Term (30)	HIE	ELISA	24 h, DOL 4, 10	1.6	Modified AKIN criteria
Sarafidis et al., 2012 [26]	Greece	Prospective case–control	Term/near term-36 GA (35)	asphyxia	ELISA	DOL 1, 3, 10	2.87	sCr based definition
El-Frargy et al., 2015 [28]	Egypt	Prospective case–control	Term (30)	critically ill	ELISA	Day 1, day 3 of admission	0.6	sCr and BUN-based renal impairment
Refat et al., 2023 [2]	Egypt	Prospective cohort study	Term (70)	asphyxia	ELISA	DOL 1	1.43	Modified KDIGO
Hidayati et al., 2021 [25]	Indonesia	Diagnostic test study	Mixed (135)	mixed NICU	PENIA	0–28 DOL	1.605	Neonatal RIFLE
Diab et al., 2022 [29]	Egypt	Prospective case–control	Preterm (90)	RDS	ELISA	DOL 3	1.25	KDIGO
Wang et al., 2020 [22]	China	Observational cross-sectional diagnostic accuracy	Term (196)	jaundice	PETIA	First 24 h after admission	1.55	KDIGO
Zhang et al., 2020 [21]	China	Case–control	term (140)	asphyxia	Latex-enhanced immunoturbidimetric assay	24 h of life	1.86	AKIN
Abdelsattar et al., 2025 [15]	Egypt	Retrospective cohort study	mixed (200)	sepsis	ELISA	First 24 h after admission	9.4	KDIGO

Abbreviations: AKI, acute kidney injury; BUN, blood urea nitrogen; ELISA, enzyme-linked immunosorbent assay; GA, gestational age; HIE, hypoxic-ischemic encephalopathy; NICU, neonatal intensive care unit; PENIA, particle-enhanced nephelometric immunoassay; PETIA, particle-enhanced turbidimetric immunoassay; pRIFLE, pediatric RIFLE criteria; RDS, respiratory distress syndrome; sCr, serum creatinine; sCysC, serum cystatin C; DOL, day of life.

### 3.3. Risk-of-Bias and Diagnostic Accuracy

**Figure 2:**
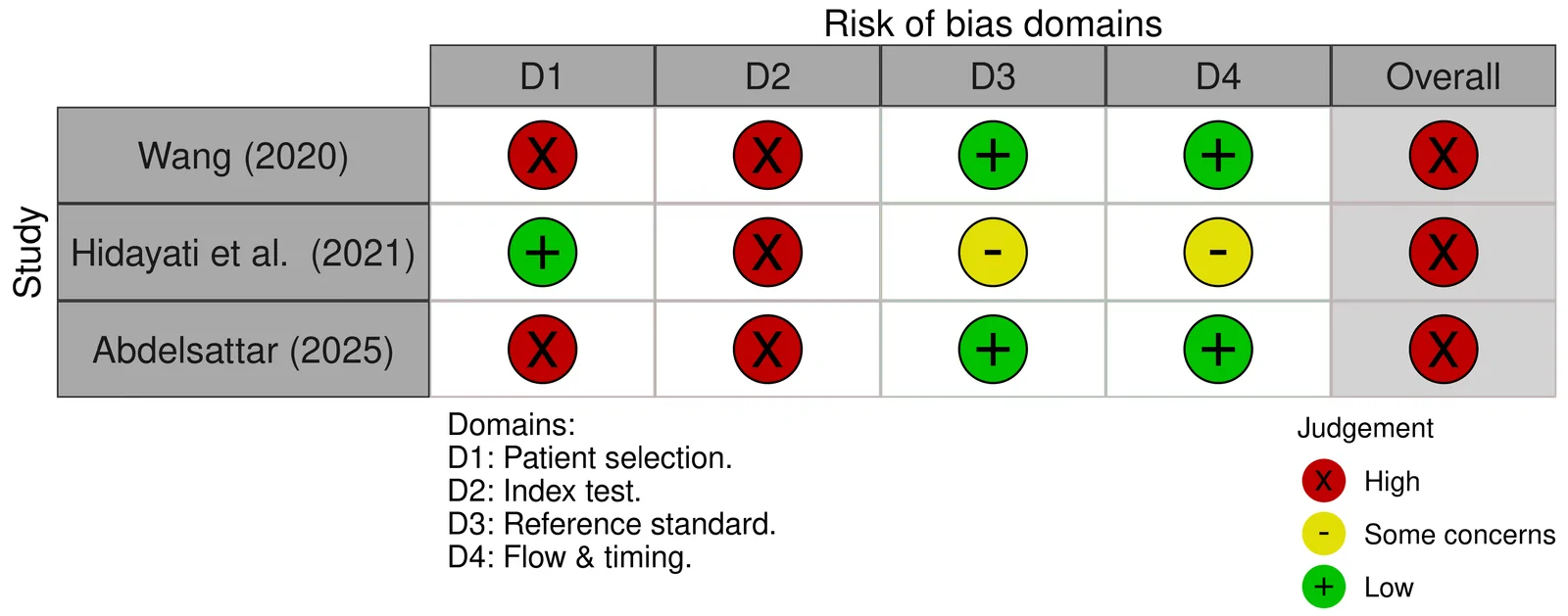
QUADAS-2 risk of bias and applicability concerns summary for the concurrent included studies across four domains (patient selection, index test, reference standard, flow and timing) [15,22,25].

**Figure 3:**
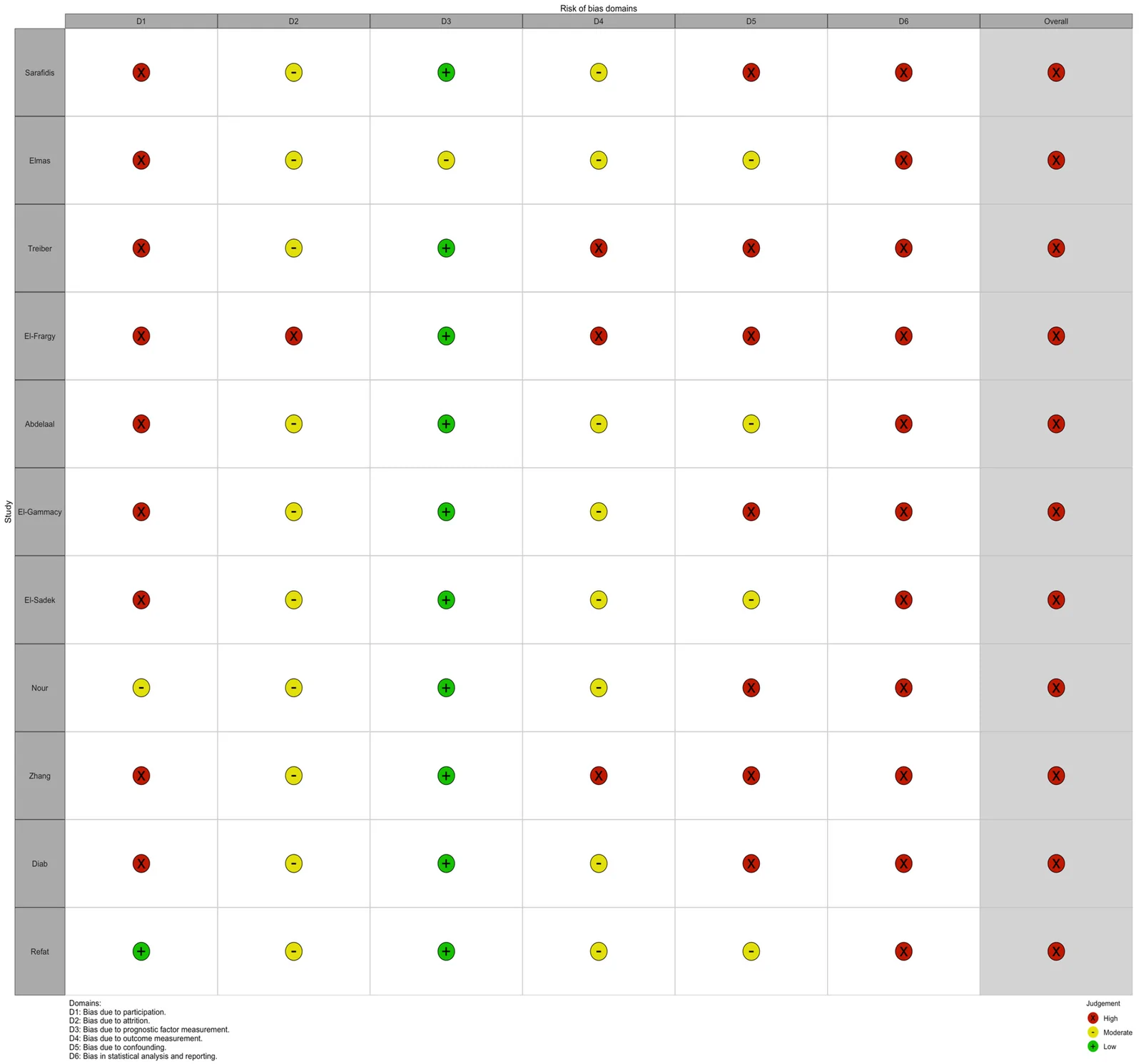
QUIPS risk-of-bias assessment of the included prognostic studies.

Risk-of-bias assessment. Risk of bias in the early predictive studies was appraised with QUIPS [19], which is designed for prognostic factor studies. PROBAST was considered and rejected because it targets multivariable prediction model studies, whereas the included studies evaluate a single marker. The three concurrent diagnostic accuracy studies were appraised with QUADAS-2. Results are summarized in Figure 2 and Figure 3.

#### 3.3.1. Risk-of-Bias Domains

QUADAS-2

Patient selection. The risk of bias was high in two studies and low in one. The main concern was the use of selected patient groups and healthy controls, which may introduce selection and spectrum bias. In contrast, one study used consecutive participant recruitment, resulting in a low risk of bias for this domain.

Index test. The risk of bias in the index test domain was high in all three studies. A notable concern was that sCysC thresholds were predominantly determined post hoc using ROC curve analysis rather than testing a pre-defined cut-off value. Therefore, none of the included studies explicitly reported blinding of index test interpretation to reference standard results.

Reference standard. The risk of bias was low in two studies and unclear in one. Two studies used KDIGO criteria, while one used nRIFLE based on creatinine-derived eGFR. Although these represent established AKI definitions, their reliance on sCr remains a limitation in neonates because of its delayed response to renal injury, maternal creatinine transfer, and gestational age dependence, potentially affecting estimates of sCysC diagnostic accuracy.

QUIPS

Bias due to participation. The risk of bias was high in 9 of 11 studies, moderate in one, and low in one. The main concern was selective recruitment, including the use of case–control designs and specific clinical populations, which may limit the representativeness of the study samples.

Bias due to confounding. The risk of bias was high in seven studies and moderate in four. Relevant neonatal and clinical factors associated with AKI were not consistently accounted for, limiting the ability to establish the independent prognostic value of sCysC.

Bias in statistical analysis and reporting. The risk of bias was high in all 11 studies. The main concerns were limited adjustment for potential confounders and data-driven estimation of predictive performance, which may reduce the robustness and generalizability of the reported associations.

Bias due to prognostic factor measurement. This domain showed the lowest risk of bias, with 10 of 11 studies rated low risk and one moderate, indicating generally adequate and consistent measurement of sCysC across studies. Bias due to attrition was rated moderate in ten studies and high in one, most often because completeness of follow-up was not clearly reported. Bias due to outcome measurement was rated moderate in eight studies and high in three. In these studies, the creatinine-based outcome was determined without blinding to the index test.

Studies were classified as early predictive when sCysC was measured at least 24 h before the creatinine-based diagnosis was established, since this is the shortest interval at which a measurement could plausibly inform a clinical decision before creatinine rises. In studies where sampling followed a fixed protocol schedule rather than a prespecified predictive design, classification followed the actual timing of measurement relative to diagnosis.

Flow and Timing. Two of the three concurrent studies showed appropriate and clearly reported intervals between sCysC measurement and sCr assessment and were rated low risk. Hidayati et al. [25] was rated as having some concerns because the interval between index test and reference standard was not clearly reported. The small number of AKI events in some studies was treated as an imprecision concern and is addressed with the sensitivity analyses, not as flow-and-timing bias.

#### 3.3.2. Applicability Concerns

Patient selection. Among the three concurrent diagnostic accuracy studies, applicability concerns for patient selection were rated low: these studies enrolled hospitalized neonates in whom the test would actually be used—critically ill, septic, or jaundiced neonates—rather than healthy controls. The predictive studies that compared sick neonates with entirely healthy controls are captured under QUIPS participation bias, where 9 of 11 studies were rated high risk precisely because such samples do not represent the at-risk population.

Index test. Applicability concerns for the index test were low across the three concurrent studies. The laboratory assays used for sCysC were validated in all three.

Reference standard. Some concerns regarding reference standard applicability were noted in one of the three concurrent studies (Hidayati et al. [25]), which used nRIFLE based on creatinine-derived eGFR; the other two used KDIGO criteria. The reliance of every reference standard on sCr remains a limitation in neonates, given its delayed response to renal injury, maternal creatinine transfer, and gestational age dependence.

#### 3.3.3. Overall Assessment

Regarding overall risk, no single study was rated low risk of bias across all domains: all three concurrent studies were rated high risk overall under QUADAS-2, and all early predictive studies were rated high risk overall under QUIPS.

The high prevalence of methodological concerns across the included studies reflects three structural problems in the existing literature. First, spectrum bias appears in several studies that used case–control designs comparing critically ill neonates with healthy controls rather than with high-risk neonates without AKI. Second, post hoc threshold selection is common: most of the included studies derived the optimal sCysC value by analyzing their own outcome data rather than testing a predefined cut-off, a practice that systematically overestimates performance. Third, and perhaps most important, is the reference standard against which sCysC was measured. Serum creatinine, used universally across the included studies, rises substantially later than sCysC. An early elevation of sCysC that precedes creatinine-defined AKI is therefore recorded as a false positive rather than recognized as an early predictive signal.

### 3.4. Sensitivity and Subgroup Analyses

As specified in the predefined review protocol, a narrative synthesis was performed, including sensitivity analysis and subgroup analysis to address the existing methodological heterogeneity.

#### 3.4.1. Sensitivity Analyses

To address potential spectrum bias, a sensitivity analysis was performed by excluding case–control studies. Six cohort-type or cross-sectional studies were retained: El-Gammacy et al. [10], Refat et al. [2], Nour et al. [23], Hidayati et al. [25], Wang et al. [22], and Abdelsattar et al. [15]. Across these studies, the predictive and diagnostic performance of sCysC remained consistently high, with AUC values from 0.844 (Wang et al. [22]) to 0.939 (Refat et al. [2]) across the five of these six studies that report one, Nour et al. [23] reporting none. This indicates that the conclusions were not artificially inflated by the case–control designs present in the literature.

In a secondary analysis, the extracted data were stratified according to the laboratory assay methodology used for sCysC measurement. ELISA-based studies reported AUC values ranging from 0.731 (Sarafidis et al. [26]) to 1.00 (Diab et al. [29]); the lower bound should be read with the caution that the Sarafidis ROC for serum cystatin C did not reach statistical significance (*p* = 0.067). Comparable results were noted in studies using automated particle-enhanced approaches, with AUC values between 0.670 (Zhang et al. [21]) and 0.97 (Abdelaal et al. [16]). These overlapping ranges should not, however, be read as demonstrating that sCysC accuracy is assay independent: assay method is almost entirely confounded with population and clinical context in this literature, so equivalence across assays cannot be isolated from these data.

A third pre-specified sensitivity analysis, excluding studies with fewer than 30 neonates, could not discriminate anything in practice: the smallest included study enrolled exactly 30 neonates, so no study was excluded and this analysis provides no information about the robustness of the findings.

#### 3.4.2. Subgroup Analysis: Term vs. Preterm Neonates

The reconstructed 2 × 2 diagnostic data (TP, FP, TN, FN), together with the corresponding study-specific sensitivity and specificity estimates, are presented in Supplementary Material Table S2. Hierarchical (bivariate random-effects) modeling was assessed for each clinically coherent subgroup; the model converged only in the RDS subgroup (four studies), yielding a pooled sensitivity of 86.8% (95% CI 38.2–98.6%) and a pooled specificity of 91.7% (95% CI 75.6–97.5%). The wide confidence interval for sensitivity reflects both the small number of studies and the outlying estimate of Elmas et al. [24]. Pooling across the full set remained infeasible because of heterogeneity in AKI definitions, assay methods, thresholds, and sampling timing.

To assess whether gestational age may modify the early predictive and diagnostic utility of sCysC, a predefined subgroup analysis comparing preterm and full-term neonates was conducted. Within the preterm subgroup, where RDS was the predominant underlying condition—the studies of Abdelaal et al. [16], El-Gammacy et al. [10], Elmas et al. [24], and Diab et al. [29]—sCysC demonstrated high early predictive accuracy, with AUC values ranging from 0.88 to 1.00. Among full-term neonates, presenting predominantly with perinatal asphyxia or HIE-Sarafidis et al. [26], Zhang et al. [21], and Refat et al. [2]—sCysC showed more variable performance, with AUC values from 0.670 to 0.939.

### 3.5. Diagnostic Accuracy Findings

Of the 13 included studies, two evaluated concurrent diagnostic accuracy only, with sCysC measured within 24 h of AKI diagnosis; ten evaluated early predictive accuracy only, with sCysC measured more than 24 h before diagnosis; and Abdelsattar et al. [15] contributed to both analyses. Table 2 summarizes the diagnostic performance of all studies by assessment timing.

(a) Concurrent Diagnostic Accuracy:

Sensitivity and specificity. Across the three concurrent diagnostic accuracy studies, sCysC sensitivity ranged from 84.8% to 88.5%, while specificity ranged from 61.8% to 75.0%. The highest sensitivity (88.46%) and specificity (75.0%) were reported by Abdelsattar et al. [15] in neonates with sepsis-associated AKI.

Area under the ROC. All three concurrent studies reported AUC values, ranging from 0.844 to 0.849, indicating good discriminatory ability (AUC ≥ 0.80). The highest AUC (0.849) was reported by Hidayati et al. [25] in critically ill newborns admitted to the NICU.

Optimal threshold values. Reported sCysC thresholds showed substantial variability. Two thresholds fell between 1.55 and 1.605 mg/L, consistent with published reference values. One study (Abdelsattar et al. [15]) reported a much higher threshold of >9.4 mg/L in newborns with sepsis-associated AKI; this outlying value may reflect the non-standardized ELISA method and a heterogeneous population with severe septic illness.

(b) Early predictive diagnostic accuracy

Sensitivity and specificity. Serum cystatin C sensitivity varied substantially across the 11 early predictive studies, ranging from 16% to 100% (Table 2). This spread reflects methodological heterogeneity as much as true variation in biomarker performance. The lowest value, 16%, reported by Elmas et al. [24] in preterm neonates with RDS at a cut-off of 1.62 mg/L, derives from a study in which only six neonates developed AKI, a number insufficient to support a reliable sensitivity estimate, and it should not be read as representative of the biomarker. Excluding it, sCysC sensitivity across the remaining studies ranged from 53.3% to 100%.

Two studies at the upper end of this range—Abdelaal et al. [16] and Diab et al. [29], both reporting 100% sensitivity—used case–control designs comparing RDS neonates with non-RDS or healthy controls, a design known to introduce spectrum bias and to overestimate discriminatory performance.

Among the studies whose comparison group was drawn from the same clinical population rather than from healthy controls, sCysC sensitivity ranged from 61.5% (Zhang et al. [21], in asphyxiated neonates without AKI) to 95% (Refat et al. [2], in a prospective cohort with perinatal asphyxia).

The reported sCysC specificity ranged from 69.3% to 100% across the same studies. Perfect specificity (100%) was reported by El-Sadek et al. [27] and Diab et al. [29]; both designs incorporated control groups without the clinical substrate of the target condition, a choice that makes it substantially easier for any biomarker to achieve high specificity by comparing fundamentally different populations. In contrast, the lowest specificity (69.3%), reported by Zhang et al. [21], came from a study whose comparison group consisted of asphyxiated neonates without AKI, a design that more accurately reflects the clinical setting in which the biomarker would be applied.

Area under the ROC. AUC values were reported by 9 of the 11 early predictive studies, ranging from 0.670 to 1.00. Seven studies indicated good-to-excellent discriminatory ability (AUC ≥ 0.80). The lowest AUC (0.670) was reported by Zhang et al. [21] in asphyxiated neonates. Two studies did not report AUC data (Nour et al. [23], El-Frargy et al. [28]).

Timing of elevation. In the early predictive studies, sCysC was elevated at scheduled sampling timepoints that preceded the creatinine-based AKI diagnosis by approximately 24 h to 4 days, the longest interval being reported by Abdelaal et al. [16] in preterm neonates with RDS. These intervals reflect each study’s sampling schedule rather than a demonstrated patient-level lead time: establishing how much earlier an individual neonate crosses a diagnostic threshold on sCysC than on sCr would require paired, patient-level timing data, which the included studies do not provide.

## 4. Discussion

This systematic review assessed the diagnostic performance of sCysC for AKI in neonates across 13 studies involving both term and preterm infants (Table 1). The evidence indicates that sCysC is a promising biomarker for neonatal AKI, showing good in several clinical settings, although with an AUC as low as 0.670 in one cohort of asphyxiated neonates, and, in the early predictive studies, elevation at scheduled timepoints earlier than the rise in sCr (Table 2).

The main finding of this review is that sCysC was elevated at sampling timepoints preceding creatinine-based AKI diagnosis by approximately 24 h to 4 days, depending on the clinical population and measurement schedule [10,16,20,23,27,28,29]. This early signal was most consistent in preterm neonates with RDS, where several studies reported high AUC values and sCysC elevation before the delayed rise in sCr. Diagnostic performance was more variable among neonates with perinatal asphyxia or HIE, suggesting that sCysC interpretation may be influenced by clinical context, illness severity, and sampling timing [2,21,23,26]. This pattern suggests that sCysC may be most clinically informative when the timing of renal vulnerability is relatively predictable, whereas its performance becomes more variable in complex multisystem conditions such as asphyxia, HIE, sepsis, and critical illness.

The findings also showed that sCysC should not be interpreted as a stand-alone replacement for sCr or established AKI criteria at this stage. Rather, the available evidence supported its potential role as an adjunctive biomarker for earlier recognition of renal dysfunction in high-risk neonates. However, heterogeneity in study populations, AKI definitions, assay methods, sampling times, and diagnostic cut-off values limited direct comparison between studies and highlighted the need for standardized neonatal thresholds before routine clinical implementation (Table 1 and Table 2).

The included studies evaluated heterogeneous neonatal populations, including preterm neonates with RDS, term neonates with perinatal asphyxia or HIE, critically ill neonates, septic neonates, and neonates with severe hyperbilirubinemia. Across these populations, the observed proportion of neonates with AKI varied substantially, from 9.7% (Elmas et al. [24]) to 66.7% (El-Frargy et al. [28]), calculated on the comparison groups used for the accuracy analyses and listed in Supplementary Material Table S2. This full-set range is not an incidence range: in the seven case–control studies, the proportion is fixed by the sampling design. Restricted to the six cohort-type studies, in which the proportion can be read as incidence, it ranged from 17.3% to 52%. This variability likely reflects differences in gestational age, underlying clinical condition, illness severity, timing of renal assessment, and AKI diagnostic criteria.

Among preterm neonates with RDS, the observed proportion with AKI ranged from 9.7% (Elmas et al. [24]) to 24.0% (Abdelaal et al. [16]), with Diab et al. [29] at 20.0% and El-Gammacy et al. [10] at 17.3%. Only El-Gammacy et al. [10] used a cohort design in this subgroup, so 17.3% is the one figure here that estimates incidence; in the other three, the proportion follows from recruitment. The highest proportions in the review were seen in critically ill and in septic neonates: 66.7% in El-Frargy et al. [28] and 52.0% in Abdelsattar et al. [15], the latter within a cohort design.

A separate line of evidence concerns the diagnostic definition itself. In a large multicenter cohort of 52,333 hospitalized neonates that was not eligible for this synthesis because it reports no diagnostic accuracy metrics against a creatinine-based reference standard, Xu et al. [13] proposed a cystatin C-based AKI definition (CyNA: sCysC ≥ 2.2 mg/L or a ≥25% increase). CyNA detected 6.5 times as many cases at elevated risk of in-hospital mortality as modified creatinine-based KDIGO criteria, and the neonates identified by CyNA alone still carried a significantly increased mortality risk. The importance of this finding is that sCysC did not merely increase the apparent AKI rate: it identified a subgroup of neonates missed by sCr-based criteria who nonetheless remained at risk, suggesting that reliance on sCr alone may underestimate the burden of neonatal AKI and challenging the assumption that sCr-based criteria should remain the sole reference framework for neonatal AKI detection.

A second report was excluded on the same design ground but bears on the question of sampling medium. Treiber et al. [20] in 100 term neonates after perinatal hypoxia or asphyxia, measured cystatin C in umbilical cord blood and on day 3 reported an AUC of 0.918. That analysis discriminates asphyxiated neonates from healthy controls rather than neonates with AKI from neonates without it, and the study reports no accuracy estimate against a creatinine-based reference standard, so it was not eligible for this synthesis and contributes no accuracy evidence to it. It is noted here only because no included study sampled cord blood, which leaves cord-blood cystatin C in the asphyxiated neonate an untested option rather than a supported one.

A central finding of this review is that sCysC diagnostic performance depended strongly on the timing of biomarker measurement in relation to AKI diagnosis. Therefore, the included studies were classified into two clinically relevant categories: concurrent diagnostic studies, in which sCysC was measured simultaneously or within the same 24 h window as AKI diagnosis, and early predictive studies, in which sCysC was measured more than 24 h before creatinine-based AKI diagnosis. This distinction is important because it separated the potential role of cystatin C as a real-time diagnostic biomarker from its potential role as an earlier predictor of AKI.

Among the studies classified as concurrent diagnostic analyses, sCysC showed good and relatively consistent performance for identifying neonatal AKI. Reported AUC values were similar across the concurrent studies, ranging from 0.844 to 0.849, with sensitivities between 84.8% and 88.46% and specificities between 61.8% and 75.0% [15,22,25]. These findings suggest that sCysC may be useful as a complementary biomarker for real-time AKI detection in critically ill neonates, neonates with hyperbilirubinemia-related AKI, and neonates with sepsis-associated AKI.

However, the moderate specificity reported in some concurrent studies indicates that elevated sCysC should not be interpreted in isolation. In the study by Hidayati et al. [25], sCysC showed high sensitivity but lower specificity when compared with sCr-based estimated GFR, supporting a role as a screening biomarker rather than a stand-alone diagnostic test. Wang et al. [22] showed that sCysC contributed to the diagnosis of hyperbilirubinemia-related AKI, particularly when combined with urinary biomarkers. In the sepsis-associated AKI study by Abdelsattar et al. [15], sCysC also showed good diagnostic performance, but interpretation may be more complex in the inflammatory context of neonatal sepsis.

Taken together, the concurrent diagnostic data suggest that sCysC performed consistently across different neonatal clinical settings, but its clinical use should be integrated with sCr, urine output, clinical condition, and other markers of illness severity.

The early predictive studies showed a wider range of diagnostic performance, with reported sCysC AUC values ranging from 0.670 to 1.00. This wider range likely reflects differences in study population, clinical context, AKI definition, assay method, timing of sampling, and cut-off derivation. Despite this heterogeneity, most early predictive studies showed that sCysC increased before sCr and could identify neonates at risk of AKI earlier than sCr-based criteria [2,10,16,21,23,24,26,27,29].

The strongest early predictive performance was observed in preterm neonates with RDS. These studies were notable for their methodological convergence: most assessed sCysC around day 3 of life, when sCr remained relatively uninformative, and several reported later creatinine elevations during the first week. Abdelaal et al. [16] reported an AUC of 0.97 for day-3 sCysC, with 100% sensitivity and 83.3% specificity at a cut-off of ≥1.28 mg/L. El-Gammacy et al. [10] similarly reported high predictive accuracy for day-3 sCysC, with an AUC of 0.92, sensitivity of 92.3% and specificity of 96% at a cut-off of >1.3 mg/L. Elmas et al. [24] and Diab et al. [29] also supported the predictive value of sCysC in this population.

By contrast, the predictive performance of sCysC was more variable in neonates with perinatal asphyxia or HIE. Zhang et al. [21] reported a lower AUC of 0.670 for sCysC measured 24 h after birth, whereas Refat et al. [2] reported high predictive accuracy at 24 h of life in a prospective cohort of asphyxiated term neonates, and Sarafidis et al. [26] reported an AUC of 0.731 on day of life 1 that did not reach statistical significance. In this clinical context, renal injury may begin antenatally or intrapartum, may be modified by resuscitation and therapeutic hypothermia, and may coexist with multiorgan dysfunction. These differences suggest that, in asphyxiated neonates, the diagnostic value of sCysC may depend particularly on the timing of sampling, severity of hypoxic injury, and associated systemic illness.

Taken together, the early predictive studies support the main hypothesis of this review: sCysC can detect neonatal renal dysfunction earlier than sCr in several high-risk populations. Nevertheless, the wide range of reported AUC values and cut-off thresholds indicated that sCysC predictive performance was not uniform across all neonatal groups and requires population-specific interpretation.

One of the most clinically relevant findings of this review is the timing pattern of sCysC relative to sCr. Across the early predictive studies, sCysC was elevated at scheduled sampling timepoints that preceded creatinine-defined AKI [10,21,23,27,28]. This was clearest in preterm neonates with RDS: Abdelaal et al. [16] reported that sCysC was significantly elevated on day 3 of life in neonates who developed AKI, whereas sCr became significantly higher only on day 7, and El-Gammacy et al. [10] and Diab et al. [29] reported that day-3 sCysC was associated with subsequent AKI before the delayed rise in sCr. In critically ill neonates, El-Sadek et al. [27] reported that plasma cystatin C increased approximately 48 h before sCr and renal resistive index changes became apparent.

Several physiological and methodological factors may explain the delayed response of sCr in neonates. Early postnatal sCr levels partly reflect maternal creatinine, limiting their accuracy in assessing newborn renal function. Serum creatinine is also a functional marker and may not increase until significant glomerular filtration loss has occurred. Interpretation is further affected by gestational age, postnatal age, renal maturation, muscle mass, hydration status, and assay method, including interference from bilirubin in colorimetric assays [25,28,30]. Neonatal renal physiology may also contribute to the timing gap between cystatin C and creatinine. In preterm and critically ill neonates, renal perfusion is highly susceptible to hemodynamic instability, hypoxemia, ductal shunting, vasoactive medications, mechanical ventilation, and nephrotoxic drugs [1,4,5]. These factors can cause early changes in glomerular filtration or tubular stress before sCr reaches diagnostic thresholds.

In contrast, sCysC appears less affected by maternal transfer and may better reflect neonatal glomerular filtration during the immediate postnatal period [4,31]. This characteristic may explain why sCysC showed earlier predictive value in several included studies. Nevertheless, cystatin C should still be interpreted within the clinical context, because its values and diagnostic thresholds may vary according to illness severity, assay method, and timing of sampling; in septic neonates, systemic inflammation may further complicate interpretation [30].

The diagnostic performance of sCysC varied across neonatal clinical contexts, suggesting that its interpretation should be population specific. The most consistent results were observed in preterm neonates with RDS. In this subgroup, sCysC showed high predictive performance, particularly when measured on day 3 of life, and several studies reported earlier elevation compared with sCrs [10,16,24,29]. This may reflect the relatively defined timing of renal vulnerability in preterm neonates with RDS, where hypoxemia, hemodynamic instability, and reduced renal perfusion may contribute to early renal dysfunction. In this setting, early respiratory failure, surfactant deficiency, hypoxemia, mechanical ventilation, hemodynamic instability, and reduced renal perfusion occur during a relatively narrow postnatal window, which may explain why day-3 cystatin C performed consistently across studies.

In neonates with perinatal asphyxia or HIE, sCysC showed more variable diagnostic performance. Zhang et al. [21] reported lower discriminatory ability for sCysC measured 24 h after birth, Refat et al. [2] reported high predictive accuracy in full-term neonates with perinatal asphyxia, and Nour et al. [23] reported a sensitivity of 86% and specificity of 75% at a cut-off of 1.6 mg/L in neonates with hypoxic-ischemic encephalopathy. This variability may be explained by differences in timing of biomarker measurement, severity of hypoxic ischemic injury, therapeutic hypothermia exposure, and the complex pattern of renal injury following asphyxia.

In septic or critically ill neonates, sCysC also showed diagnostic value, but interpretation may be more complex. Hidayati et al. [25] found that sCysC had good sensitivity but only moderate specificity in critically ill neonates, supporting its use as a screening biomarker rather than a stand-alone diagnostic test. Abdelsattar et al. [15] reported good diagnostic performance in sepsis-associated AKI, although systemic inflammation may influence sCysC levels and should be considered when interpreting results in septic neonates. El-Sadek et al. [27] also supported the early predictive value of plasma cystatin C in critically ill neonates, reporting earlier elevation compared with sCr and renal resistive index.

Among neonates with severe hyperbilirubinemia, Wang et al. [22] reported that sCysC contributed to the early diagnosis of hyperbilirubinemia-related AKI, particularly when combined with urinary biomarkers such as uNGAL, KIM-1, and TIMP-2. This finding suggests that sCysC may be useful in clinical settings where sCr interpretation may be affected by assay-related limitations. However, the evidence in this subgroup is limited to a small number of studies, and further validation is needed before firm conclusions can be drawn.

A major limitation across the included studies was the heterogeneity of sCysC cut-off values. Ten of the thirteen reported diagnostic thresholds fell between 1.25 and 2.68 mg/L, the remaining three being 0.60, 2.87, and 9.40 mg/L, but the optimal cut-off varied according to population, timing of measurement, assay method, and AKI definition [10,16,22,25,27]. For example, in preterm neonates with RDS, Abdelaal et al. [16] reported a day-3 cut-off of ≥1.28 mg/L, while El-Gammacy et al. [10] reported a similar cut-off of 1.3 mg/L. In contrast, El-Sadek et al. [27] reported a higher cut-off of 2.68 mg/L in critically ill neonates, suggesting that thresholds derived in one clinical population may not be directly applicable to another.

This variability was further increased by differences in assay methodology. Across the included studies, sCysC was measured using different laboratory methods, including ELISA, PENIA, PETIA, and other laboratory-based approaches. Because sCysC values may vary between assays, differences in measurement technique can affect both absolute values and derived diagnostic thresholds [15,16,24,25].

Another important source of heterogeneity was the use of post hoc ROC-derived sCysC thresholds. In most studies, the optimal cut-off value was selected after data analysis rather than predefined before patient enrollment. Although this approach is useful for exploratory biomarker research, it may overestimate diagnostic performance and limited external validity. Few studies validated their proposed thresholds in an independent cohort. Therefore, the high AUC values reported in some studies, particularly small single-center or case–control studies, should be interpreted cautiously [2,10,16,29].

The outlying sCysC threshold reported by Abdelsattar et al. [15] in sepsis-associated AKI should also be interpreted with caution. This higher cut-off may reflect differences in assay method, study population, inflammatory status, or the specific diagnostic framework used in that study, rather than a generalizable sCysC threshold for neonatal AKI [15]. The current evidence does not support the use of a single universal sCysC cut-off for all neonates. Instead, future studies should aim to validate assay-specific, gestational-age-specific, and population-specific thresholds before sCysC can be routinely incorporated into neonatal AKI diagnostic pathways.

The optimal timing of sCysC measurement appears to be context dependent rather than universal. Across the included studies, its diagnostic value was strongly influenced by the timing of measurement; the optimal sampling window appeared to differ according to neonatal population and clinical context.

In preterm neonates with RDS, day 3 of life emerged as the most consistent and clinically useful measurement point. Abdelaal et al. [16] reported that sCysC measured on day 3 predicted AKI earlier than sCr, which became significantly elevated only later in the first week. El-Gammacy et al. [10] found that day-3 sCysC predicted AKI in preterm neonates with RDS, and Diab et al. [29] reported perfect discrimination for day-3 sCysC in the same population. Elmas et al. [24] further supported early postnatal sCysC assessment. These findings suggest that day 3 may represent a useful balance between postnatal renal adaptation and early detection of evolving kidney injury in preterm neonates with RDS.

In neonates with perinatal asphyxia or HIE, earlier sampling may be more informative. Zhang et al. [21] assessed sCysC at 24 h after birth, and Refat et al. [2] supported the early predictive value of sCysC measured on the first day of life in full-term neonates with perinatal asphyxia. These findings suggest that, in asphyxiated neonates, sampling during the first 24 h of life may better capture early renal dysfunction related to hypoxic ischemic injury.

For critically ill neonates, the available evidence supports the value of early sampling at admission or within the first days of clinical deterioration, but the optimal timing remains less clearly defined. El-Sadek et al. [28] reported that plasma cystatin C increased approximately 48 h before sCr and renal resistive index changes, while Hidayati et al. [25] evaluated sCysC as a concurrent diagnostic biomarker in critically ill neonates. In neonates with HIE undergoing or not undergoing therapeutic hypothermia, Nour et al. [23] used serial measurements and showed that ongoing kidney injury may persist despite improvement in sCr and urine output. These findings suggest that serial monitoring may be more informative than a single measurement in unstable or critically ill neonates.

The available evidence suggests that sCysC measurement should be adapted to the clinical context: day 3 of life may be most useful in preterm neonates with RDS, sampling within the first 24 h of life may be more appropriate after perinatal asphyxia, and serial monitoring may be preferable in critically ill neonates or those with evolving multiorgan dysfunction. However, these proposed timing windows require prospective validation before they can be translated into standardized neonatal AKI protocols.

The findings of this review are consistent with the previous neonatal biomarker literature showing that sCr-based AKI diagnosis is limited and that alternative biomarkers may support earlier recognition of renal dysfunction [32,33]. Kuo et al. [32] found that several biomarkers, including sCysC, have been studied in premature infants, but meta-analysis was feasible only for urinary NGAL because many biomarkers were reported by single studies. This supports the interpretation that evidence on neonatal AKI biomarker remains promising but heterogeneous and highlights the value of a cystatin C-focused synthesis.

The early rise of sCysC before creatinine-based AKI diagnosis in the present review is also consistent with concerns regarding sCr in neonates [11,33]. In the included studies, sCysC preceded sCr-based AKI diagnosis by approximately 24 h to 4 days, especially in preterm neonates with RDS and in some neonates exposed to perinatal hypoxia or asphyxia [10,11,16,27,29]. Borloo et al. [11] showed that sCr trended in neonates undergoing whole-body hypothermia after perinatal asphyxia differ from reference patterns, supporting the difficulty of creatinine-based AKI assessment in this setting.

The more variable performance observed in asphyxia and HI may reflect differences in disease severity, timing of sampling, therapeutic hypothermia exposure, systemic illness, and creatinine kinetics [11,33]. By contrast, preterm neonates with RDS showed more consistent early predictive performance across the included studies [10,16,24,29].

Evidence from sick neonates further supports the plausibility of sCysC as a renal function marker. Avong et al. [14] found that sCysC-derived estimated GFR approximated an inulin reference more closely than creatinine-derived estimates in sick neonates, although this concerns GFR estimation rather than AKI diagnostic accuracy. The findings of Xu et al. [13] sit alongside this evidence in challenging the assumption that sCr-based definitions should remain the only reference framework for neonatal AKI detection: in that large multicenter cohort, cystatin C-based criteria identified more neonates at increased risk of in-hospital mortality than modified KDIGO creatinine-based criteria, suggesting that the latter may miss clinically relevant renal dysfunction during the neonatal period.

A key contribution of this review is distinguishing concurrent diagnostic accuracy from early predictive accuracy. This distinction may help explain differences across studies and is clinically relevant because sCysC measured at AKI diagnosis and sCysC measured before sCr-based AKI diagnosis represent different biomarker applications [32,33].

The evidence supports sCysC as a promising adjunctive biomarker for neonatal AKI, but not as a replacement for sCr or established AKI criteria. Heterogeneity in population, clinical setting, AKI definition, assay method, sampling time, and threshold derivation limits certainty; therefore, predefined, assay-specific, and population-specific thresholds require validation before routine clinical implementation.

This review has several strengths. First, it focuses specifically on sCysC as a diagnostic and early predictive biomarker for neonatal AKI, a population in which sCr has important physiological and methodological limitations. Second, the review covered different neonatal clinical contexts—preterm neonates with RDS, neonates with perinatal asphyxia or HIE, critically ill and septic neonates, and neonates with severe hyperbilirubinemia—and addressed these settings separately, allowing a clinically relevant comparison across high-risk populations. Third, the review distinguished concurrent diagnostic accuracy from early predictive accuracy, which reflect different clinical uses of cystatin C, and applied a risk-of-bias tool matched to each design: QUADAS-2 and QUIPS, respectively.

However, several limitations should be considered when interpreting the findings. The included studies were heterogeneous in terms of population characteristics, gestational age, illness severity, AKI definitions, timing of biomarker measurement, assay methods, and diagnostic thresholds. This heterogeneity limits direct comparison between studies and makes it difficult to define a single sCysC cut-off applicable to all neonates. Most studies also used post hoc ROC-derived cut-off values rather than predefined thresholds, which may overestimate diagnostic accuracy and reduce external validity.

Also, several studies had relatively small sample sizes, single-center designs, or case–control methodology. These design features may increase the risk of selection bias and may partly explain the very high AUC values reported in some subgroups, particularly among preterm neonates with RDS [10,16,24,29]. By contrast, larger and more heterogeneous studies, such as those including critically ill neonates or broader hospitalized neonatal populations, reported more moderate or more variable diagnostic performance [13,25].

Another important limitation is that sCr-based AKI criteria were frequently used as the reference standard. Because sCr itself is delayed and physiologically problematic in the neonatal period, diagnostic accuracy estimates for sCysC may be affected by imperfections in the comparator. Therefore, some apparent false-positive sCysC results may in fact represent renal dysfunction not captured by delayed sCr-based criteria. This is particularly relevant during the first days of life, when sCr may reflect maternal levels and may not accurately represent neonatal renal function. Finally, the lack of standardized neonatal sCysC reference intervals, assay-specific calibration, and gestational-age-specific thresholds limited the immediate clinical applicability of the findings.

The findings of this review support the potential clinical value of sCysC as an adjunctive biomarker for earlier recognition of AKI in high-risk neonates. Its main clinical advantage is the possibility of detecting renal dysfunction before sCr becomes abnormal, particularly in preterm neonates with RDS and in neonates exposed to perinatal hypoxia or asphyxia [10,16,29]. Earlier identification of neonates at risk of AKI may allow closer monitoring of renal function, optimization of fluid balance, avoidance or adjustment of nephrotoxic medications, and earlier consideration of nephrology consultation.

However, the current evidence does not support replacing sCr or established current AKI criteria with sCysC alone. Instead, sCysC should be considered a complementary biomarker that may improve early risk stratification when interpreted alongside sCr, urine output, gestational age, postnatal age, clinical condition, and exposure to nephrotoxic or hemodynamically significant events. Clinically, sCysC is more likely to be useful as part of a risk-stratification strategy or serial monitoring pathway than as a single isolated diagnostic threshold. This is particularly important since sCysC performance varied across clinical settings and diagnostic thresholds were not uniform between studies [13,15,22,25].

Future research should focus on prospective, adequately powered, multicenter studies using standardized sCysC assays and predefined diagnostic thresholds. Separate validation is needed for term and preterm neonates, as well as for specific clinical conditions such as RDS, perinatal asphyxia, sepsis, hyperbilirubinemia, and mixed critically ill neonatal cohorts. Future studies should also evaluate whether sCysC-guided monitoring improves clinically relevant outcomes, including progression of AKI, need for renal replacement therapy, duration of mechanical ventilation, length of NICU stay, mortality, and long-term kidney function. Future research should also assess whether sCysC-guided clinical decisions, not merely sCysC measurement, improve neonatal outcomes.

Another important research priority is the development of assay-specific and gestational-age-specific neonatal reference intervals. Because several included studies used post-hoc thresholds, future studies should test sCysC predefined cut-offs in independent cohorts rather than deriving thresholds from the same population in which diagnostic accuracy is assessed. This would improve external validity and help determine whether sCysC can be incorporated into standardized neonatal AKI diagnostic algorithms.

## 5. Conclusions

In conclusion, sCysC is a promising adjunctive biomarker for early detection of neonatal AKI. Most studies reported good diagnostic performance, with sCysC becoming abnormal at earlier scheduled timepoints than sCr. The most consistent evidence comes from preterm neonates with RDS. In perinatal asphyxia, HIE, sepsis, critical illness, and hyperbilirubinemia-related AKI, sCysC also showed diagnostic potential, though results varied with clinical context, sampling time and study methodology.

Whether earlier biochemical detection translates into earlier intervention and better outcomes was not tested by any included study, and it remains the key question for prospective trials. At present, sCysC should be used as a complementary biomarker rather than as a replacement for sCr or established AKI criteria. Routine clinical use is limited by the absence of standardized assay methods, validated neonatal reference intervals, and population-specific diagnostic thresholds. Prospective multicenter studies are required to validate sCysC cut-offs and to assess whether sCysC-guided monitoring improves neonatal outcomes.

## Figures and Tables

**Table 2:** Diagnostic performance of serum cystatin C across included studies based on time-related design.

Study (Author, Year)	n (AKI/Total Enrolled)	AUC	Sensitivity (%)	Specificity (%)	Cut-Off (mg/L)	Interval Between Scheduled sCysC and sCr Measurements
Sarafidis et al., 2012 [26] *	8/35	0.731	66.7	88.5	2.87	sCysC at 24 h; sCr at 48–72 h
Elmas et al., 2013 [24] *	6/62	0.88	16	82	1.62	sCysC at 72 h; sCr within subsequent 72 h
El-Frargy et al., 2015 [28] *	20/30	NR	85	80	0.6	sCysC during first 24 h; sCr at 72 h
Abdelaal et al., 2017 [16] *	24/100	0.97	100	83.3	≥1.28	sCysC at 72 h; sCr on DOL 7
El-Gammacy et al., 2018 [10] *	13/75	0.92	92.3	96	1.3	sCysC at 72 h; sCr on DOL 5
El-Sadek et al., 2020 [27] *	30/90	0.804	53.3	100	2.68	sCysC at 24 h; sCr at 72 h
Nour et al., 2020 [23] *	14/30	NR	86	75	1.6	sCysC at 24 h; sCr at 48 h
Zhang et al., 2020 [21] *	37/140	0.67	61.5	69.3	1.86	sCysC at 24 h; sCr not reported
Diab et al., 2022 [29] *	18/90	1.00	100	100	1.25	sCysC at 72 h; sCr on DOL 5
Refat et al., 2023 [2] *	21/70	0.939	95	94	1.43 ^	sCysC at 24 h; sCr at 48–72 h
Wang et al., 2020 [22] **	27/196	0.844	85.2	62.9	1.55	sCysC first 24 h; sCr not reported
Hidayati et al., 2021 [25] **	32/135	0.849	84.8	61.8	1.605	NR
Abdelsattar et al., 2025 [15] ***	52/200	0.848	88.46	75.0	9.4	Precedes or concurrent (days 0, 2, 4)

Legend: * early predictive study; ** concurrent study; *** both early predictive and concurrent study; AKI, acute kidney injury; AUC, area under the curve; NR, not reported; values indicate the scheduled sampling timepoints in each study, not a demonstrated lead time at patient level. The n column gives AKI cases over total neonates enrolled. For Wang et al. [22] and Abdelsattar et al. [15], the diagnostic accuracy analysis was performed within a subgroup rather than the full enrolled cohort (27 AKI versus 35 non-AKI, and 52 AKI versus 48 non-AKI, respectively); the denominators actually used for sensitivity and specificity in every study are given in Supplementary Material Table S2, and all proportions quoted in the text are calculated on those denominators. NB: ^ Refat et al. [2] print the cut-off as 1.43 ng/mL. The unit is a misprint in the source: the study’s own cystatin C concentrations are reported on the mg/L scale and are compared directly with mg/L reference values, so the cut-off is presented here as 1.43 mg/L.

## Data Availability

All data extracted and analyzed for this systematic review are included in the article and its Supplementary Materials.

## Supplementary Materials

The following supporting information can be downloaded at https://www.mdpi.com/article/10.3390/children13091202/s1: Supplementary Material File S1—PRISMA-DTA checklist; Supplementary Material File S2—SWiM checklist; Supplementary Material File S3—Complete search strategies for all four databases; Supplementary Material Table S1—Excluded studies with reasons; Supplementary Material Table S2—Reconstructed 2 × 2 diagnostic data; Supplementary Material Table S3—Funding sources reported by the included studies. The protocol is openly accessible in PROSPERO (CRD420261302574) starting 8 February 2026 at https://www.crd.york.ac.uk/prospero.

## Abbreviations

The following abbreviations are used in this manuscript:

AKI	acute kidney injury
AKIN	Acute Kidney Injury Network
AUC	area under the receiver operating characteristic curve
eGFR	estimated glomerular filtration rate
ELISA	enzyme-linked immunosorbent assay
GA	gestational age
GFR	glomerular filtration rate
HIE	hypoxic-ischemic encephalopathy
KDIGO	Kidney Disease: Improving Global Outcomes
NICU	neonatal intensive care unit
PENIA	particle-enhanced nephelometric immunoassay
PETIA	particle-enhanced turbidimetric immunoassay
PRISMA-DTA	Preferred Reporting Items for Systematic Reviews and Meta-Analyses for Diagnostic Test Accuracy
pRIFLE	pediatric Risk, Injury, Failure, Loss, End-Stage Renal Disease
PROSPERO	International Prospective Register of Systematic Reviews
QUADAS-2	Quality Assessment of Diagnostic Accuracy Studies 2
RDS	respiratory distress syndrome
ROC	receiver operating characteristic
sCr	serum creatinine
sCysC	serum cystatin C
SWiM	Synthesis Without Meta-analysis
