# Supplementary material for: Serum Cystatin C as an Early Predictive Biomarker for Acute Kidney Injury (AKI) in Neonates: A PRISMA-DTA Systematic Review
Source: Children (Basel). 2026 Sep 5;13(9):1202. doi: 10.3390/children13091202 (PMC13605153; doi:10.3390/children13091202)
Supplement: Supplementary file 1 [file children-13-01202-s001.zip › Supplementary Material File S1_PRISMA-DTA-checklist_2026-08-25.pdf]

Supplementary Material S1. PRISMA-DTA checklist

Serum cystatin C as an early predictive biomarker for acute kidney injury (AKI) in neonates: a PRISMA-DTA systematic review. Manuscript children-4488016.

Items follow the PRISMA-DTA statement (McInnes et al., JAMA 2018;319:388-396), reference [31] of the manuscript. Locations are given as manuscript sections rather than page numbers, because section anchors survive typesetting; page numbers can be inserted from the proof if the journal requires them.

Section and item	No	Checklist item	Location in this manuscript
**TITLE**<br>**Title**	1	Identify the report as a systematic review (with or without meta-analysis) of diagnostic test accuracy studies.	Title page. The title reads "... a PRISMA-DTA systematic review".
**ABSTRACT**<br>**Abstract**	2	Structured abstract covering background, aim, methods, results and conclusion.	Abstract.
**INTRODUCTION**<br>**Rationale**	3	Describe the rationale for the review in the context of what is already known.	Section 1, Introduction, paragraphs 1 to 5.
**Clinical role of index test**	4	State the scientific and clinical background, including the intended use and clinical role of the index test.	Section 1, Introduction, paragraphs 4 to 6; the intended role as an adjunctive early marker is stated and revisited in Section 4.
**Objectives**	5	Provide an explicit statement of the questions addressed, in terms of participants, index test and target condition.	Section 1, Introduction, final two paragraphs.
**METHODS**<br>**Protocol and registration**	6	Indicate whether a protocol exists, where it can be accessed, and give the registration number.	Section 2 A. PROSPERO CRD420261302574.
**Eligibility criteria**	7	Specify study and report characteristics used as eligibility criteria, with rationale.	Section 2 B, criteria a to e.
**Information sources**	8	Describe all information sources and the date each was last searched.	Section 2 C(a). PubMed/MEDLINE 1 February 2026, Cochrane CENTRAL 2 February 2026, Web of Science 2 February 2026, Scopus 8 March 2026.
**Search**	9	Present the full search strategy for every database, including any limits, so that it could be repeated.	Section 2 C(b) and C(c); full strings in Supplementary Material 3.
**Study selection**	10	State the process for selecting studies at screening and at eligibility.	Section 2 D. Two independent reviewers in Rayyan, blinded to each other, third reviewer for disagreements.
**Data collection process**	11	Describe the method of data extraction and any process for confirming data with investigators.	Section 2 D, second and third paragraphs. Standardized form, piloted on a subset, duplicate independent extraction.
**Definitions for data extraction**	12	Provide the definitions used for the target condition, index test, reference standard and other characteristics.	Section 2 B for the target condition, index test and reference standard; Section 2 E for the timing classification that separates concurrent from early predictive designs.
**Risk of bias and applicability**	13	Describe the methods used to assess risk of bias and concerns about applicability.	Section 2 F. QUADAS-2 for the concurrent diagnostic studies, QUIPS for the early predictive studies, with the reason for using two tools given in Section 3.3.
**Diagnostic accuracy measures**	14	State the principal accuracy measures and the unit of assessment.	Section 2 B(e) and Section 2 G. Sensitivity, specificity and AUC, per patient.
**Synthesis of results**	15	Describe how data were handled and combined, including multiple target-condition definitions, multiple positivity thresholds and different reference standards.	Section 2 G. Structured narrative synthesis under SWiM; the sources of heterogeneity that prevented pooling are listed there and revisited in Section 3.4.
**Meta-analysis**	16	Report the planned method of obtaining summary measures, if meta-analysis was performed.	Section 2 G and Section 3.4.2. No meta-analysis across the full set. A bivariate random-effects model was attempted per clinically coherent subgroup and converged only in the RDS subgroup; that single pooled estimate is reported with its confidence intervals.
**Additional analyses**	17	Describe the methods of any additional analyses and indicate which were pre-specified.	Section 2 G, Sensitivity and Subgroup Analyses. Three pre-specified sensitivity analyses and one pre-specified subgroup analysis.
**RESULTS**<br>**Study selection**	18	Give the numbers screened, assessed and included, with reasons for exclusion at each stage, ideally with a flow diagram.	Section 3.1 and Figure 1. Reasons for each full-text exclusion in Supplementary Material Table 1.

**Study characteristics**	19	For each included study give the citation and key characteristics, including participants, setting, design, target-condition definition, index test, reference standard, sample size and funding source.	Section 3.2 and Table 1 for items a to g; funding sources for every included study in Supplementary Material Table 3.
**Risk of bias and applicability**	20	Present the risk-of-bias and applicability assessment for each study.	Section 3.3 with subsections 3.3.1 to 3.3.3; Figure 2 (QUADAS-2) and Figure 2A (QUIPS).
**Results of individual studies**	21	For each analysis report the 2x2 data with accuracy estimates and confidence intervals, ideally with a forest or ROC plot.	Table 2 gives the published accuracy estimates per study. Reconstructed 2x2 cells, with the denominator used for each study, are in Supplementary Material Table 2. No forest or summary ROC plot is presented because the data were not pooled across the full set; the reason is stated in Section 2 G.
**Synthesis of results**	22	Describe test accuracy including variability; if meta-analysis was done, give results with confidence intervals.	Section 3.5, reported separately for the concurrent and the early predictive groups; the one pooled subgroup estimate is in Section 3.4.2.
**Additional analyses**	23	Give the results of any additional analyses.	Section 3.4.1 for the three sensitivity analyses, including the one that could not discriminate; Section 3.4.2 for the term versus preterm subgroup analysis.
**DISCUSSION Summary of evidence**	24	Summarize the main findings and the strength of the evidence.	Section 4, opening paragraphs, and Section 4 paragraphs on concurrent and early predictive accuracy.
**Limitations**	25	Discuss limitations of the included studies and of the review process.	Section 4, the strengths paragraph followed by four limitation paragraphs covering heterogeneity, post-hoc thresholds, design features and the imperfect reference standard.
**Conclusions**	26	Give a general interpretation in the context of other evidence, and discuss implications for practice and research, including the intended use and clinical role of the index test.	Section 4, clinical implications and future research paragraphs; Section 5, Conclusions.
**FUNDING Funding**	27	Describe the sources of funding for the systematic review and the role of the funders.	Funding statement. This research received no external funding.

Note on items 16 and 21. This review is a structured narrative synthesis. No summary ROC or forest plot is presented across the full set of studies because the studies differ in target-condition definition, assay, positivity threshold and sampling timing to a degree that the review judged incompatible with pooling; the reasoning is given in Section 2 G and the consequences are discussed in Section 4. A bivariate random-effects model was fitted per clinically coherent subgroup and converged only in the respiratory distress syndrome subgroup, where the pooled estimate is reported with its confidence intervals in Section 3.4.2.
