# Supplementary material for: Serum Cystatin C as an Early Predictive Biomarker for Acute Kidney Injury (AKI) in Neonates: A PRISMA-DTA Systematic Review
Source: Children (Basel). 2026 Sep 5;13(9):1202. doi: 10.3390/children13091202 (PMC13605153; doi:10.3390/children13091202)
Supplement: Supplementary file 1 [file children-13-01202-s001.zip › Supplementary Material File S2_SWiM-checklist_2026-08-25.pdf]

Supplementary Material S2. SWiM checklist

Serum cystatin C as an early predictive biomarker for acute kidney injury (AKI) in neonates: a PRISMA-DTA systematic review. Manuscript children-4488016.

Items follow the Synthesis Without Meta-analysis reporting guideline (Campbell et al., BMJ 2020;368:l6890), reference [32] of the manuscript. SWiM was developed for reviews of intervention effects; each item below is answered in the diagnostic test accuracy sense, and where an item does not transfer cleanly the answer says so rather than forcing a fit. Locations are given as manuscript sections rather than page numbers, so that they survive typesetting.

Section	No	SWiM item	How and where it is reported
**Methods**	1	Grouping studies for synthesis. Describe which studies are grouped for each synthesis and how the grouping was decided.	Section 2 E and Section 2 G. Studies were grouped on the timing between the index test and the reference standard: concurrent diagnostic accuracy when sCysC was measured within the same 24-hour window as the creatinine-based AKI diagnosis, and early predictive accuracy when it was measured more than 24 hours before. That grouping was pre-specified in the PROSPERO protocol and is the review question itself, since the two designs answer different clinical questions. Within the early predictive group, studies were further grouped by clinical context (RDS, perinatal asphyxia or HIE, sepsis, critical illness, hyperbilirubinemia) for the subgroup analysis, and by gestational age for the pre-specified term versus preterm comparison.
**Methods**	2	Standardised metric and transformation methods. Describe the standardised metric used for each outcome and any transformation applied.	Section 2 B(e) and Section 2 G. The accuracy metrics are sensitivity, specificity and the area under the ROC curve, each per patient, taken as published by the source study. No transformation was applied to any published estimate. Where a 2x2 table was needed and none was published, integer cells were reconstructed from the published sensitivity, specificity and group sizes; the reconstruction is reported in Supplementary Material Table 2 together with the denominator used for each study and with the deviation between each reconstructed and published figure.
**Methods**	3	Synthesis methods. Describe and justify the methods used to synthesise the results.	Section 2 G. A structured narrative synthesis was used. Statistical pooling across the full set was judged inappropriate because the studies differ in the definition of the target condition, in assay method, in positivity threshold and in sampling timing, and the review treats sampling timing as the variable of interest rather than as noise. A bivariate random-effects model was fitted within each clinically coherent subgroup and converged only in the RDS subgroup, where the pooled estimate is reported with confidence intervals (Section 3.4.2). Results are otherwise reported study by study and grouped as described in item 1.
**Methods**	4	Criteria used to prioritise results. Give the criteria used to select particular studies or results for the main synthesis or for drawing conclusions.	Section 2 G and Section 3.4.1. No study was given priority over another on the basis of its result. Two design criteria are applied transparently and in both directions: case-control studies with healthy control groups were flagged for spectrum bias and removed in a pre-specified sensitivity analysis, and estimates derived from very small numbers of AKI events are identified as imprecise in the text rather than dropped. Where a source qualifies its own estimate, that qualification is carried into this review, as with the non-significant ROC analysis of Sarafidis et al. ($p = 0.067$).
**Methods**	5	Investigation of heterogeneity. State the methods used to examine heterogeneity in the reported effects.	Section 2 G and Section 3.4. Heterogeneity was examined narratively and through three pre-specified sensitivity analyses (exclusion of case-control designs, stratification by assay method, exclusion of studies enrolling fewer than 30 neonates) and one pre-specified subgroup analysis (term versus preterm). The third sensitivity analysis could not discriminate in practice, because the smallest included study enrolled exactly 30 neonates; Section 3.4.1 reports that rather than omitting the analysis. The assay stratification is reported with the explicit caveat that assay method is almost entirely confounded with population and clinical context in this literature.
**Methods**	6	Certainty of evidence. Describe the methods used to assess certainty in the synthesis findings.	Section 2 F and Section 3.3. Certainty was approached through risk of bias assessed with the tool matched to each design, QUADAS-2 for the concurrent diagnostic studies and QUIPS for the early predictive studies, with signalling questions adapted to neonatal AKI and with applicability concerns rated separately. GRADE was not applied.

			Section 3.3.3 states the overall assessment and Section 3.3 the three structural problems that drive it: spectrum bias, post-hoc threshold selection and an imperfect creatinine-based reference standard.
**Methods**	7	Data presentation methods. Describe the graphical and tabular methods used to present the results.	Table 1 gives the characteristics of every included study, Table 2 the published accuracy estimates grouped by timing design, and Supplementary Material Table 2 the reconstructed 2x2 data with denominators. Figure 1 is the PRISMA-DTA flow diagram, Figure 2 the QUADAS-2 summary and Figure 2A the QUIPS assessment. No forest plot or summary ROC curve is presented across the full set, because the effects were not pooled there; the reason is given in Section 2 G.
**Results**	8	Reporting results. For each synthesis, describe the results, including the direction and size of effect and any uncertainty.	Section 3.5 reports the concurrent and the early predictive groups separately, each with the range of sensitivity, specificity and AUC and with the studies at each bound named. Section 3.4 reports the sensitivity and subgroup analyses, including the one pooled estimate with its confidence intervals. Ranges quoted in the text are calculated on the denominators listed in Supplementary Material Table 2, and the legend of Table 2 states where those differ from the enrolled totals.
**Discussion**	9	Limitations of the synthesis. Report the limitations of the synthesis methods and of the groupings used.	Section 4, limitations paragraphs. The synthesis is narrative, so it produces no summary estimate for most groupings and no formal measure of between-study heterogeneity. The groupings themselves rest on the timing of sampling as reported by each study, which is a sampling schedule rather than a measured interval between biomarker rise and diagnosis; the manuscript states this in Section 2 E, in Section 3.5 and in the legend of Table 2 rather than once. The reference standard is creatinine-based and is itself delayed in neonates, so accuracy estimates for sCysC are limited by the comparator. Seven of the thirteen studies are case-control, so proportions with AKI in those studies are set by recruitment and estimate no incidence, which is why the Discussion reports the cohort-only range alongside the full-set range.
