# Supplementary material for: Serum Cystatin C as an Early Predictive Biomarker for Acute Kidney Injury (AKI) in Neonates: A PRISMA-DTA Systematic Review
Source: Children (Basel). 2026 Sep 5;13(9):1202. doi: 10.3390/children13091202 (PMC13605153; doi:10.3390/children13091202)
Supplement: Supplementary file 1 [file children-13-01202-s001.zip › Supplementary Material File S3_Search-strategies_2026-08-25.pdf]

Database	PubMed (MEDLINE)	Cochrane CENTRAL	WoS	Scopus
**Date of search**	1 February 2026	2 February 2026	2 February 2026	8 March 2026
**Search string**	("Cystatin C"[Mesh] OR "cystatin C"[tiab] OR "cystatin-C"[tiab])<br><br>AND<br><br>("Infant, Newborn"[Mesh] OR neonat*[tiab] OR newborn*[tiab] OR preterm[tiab] OR NICU[tiab])<br><br>AND<br><br>("Acute Kidney Injury"[Mesh] OR "acute kidney injury"[tiab] OR AKI[tiab] OR "acute renal failure"[tiab])	(neonate* OR newborn* OR neonatal) AND ("cystatin C" OR cystatin-c OR "serum cystatin C") AND ("acute kidney injury" OR AKI OR "acute renal failure")	(cystatin c OR cystatin-c) AND Topic: (acute kidney injury OR AKI OR acute renal failure) AND Topic: (neonate* OR newborn* OR preterm*)	TITLE-ABS-KEY(<br>neonat* OR newborn* OR infant* OR preterm* OR prematur*<br>OR "low birth weight" OR LBW OR VLBW OR "very low birth weight"<br>OR ELBW OR NICU<br>)<br>AND TITLE-ABS-KEY(<br>"cystatin c" OR "cystatin-c" OR cysc OR scysc<br>OR "serum cystatin" OR cst3 OR "cystatin 3"<br>)<br>AND TITLE-ABS-KEY(<br>"acute kidney injury" OR AKI OR "acute renal failure"<br>OR "acute renal insufficiency"

				OR "acute kidney failure" OR "kidney injur*" OR "renal injur*" OR KDIGO OR AKIN OR pRIFLE OR RIFLE)

Search dates are written out in full to avoid any day/month ambiguity and match the dates reported in Section 2, C(a) of the manuscript. No language, date or study-design filters were applied at the search stage.
