# Supplementary material for: Serum Cystatin C as an Early Predictive Biomarker for Acute Kidney Injury (AKI) in Neonates: A PRISMA-DTA Systematic Review
Source: Children (Basel). 2026 Sep 5;13(9):1202. doi: 10.3390/children13091202 (PMC13605153; doi:10.3390/children13091202)
Supplement: Supplementary file 1 [file children-13-01202-s001.zip › Supplementary-Table-S1_Excluded-studies_2026-08-25.pdf]

Study ID	*Identification Source*	*Reason for Exclusion*
Ahmed et al. (2019)	Database Search	**Lacks mandatory diagnostic accuracy metrics.** The study compares means but does not report AUC-ROC, Sensitivity, or Specificity [Ahmed 2019].
Savrun et al. (2017)	Database Search	**Lacks mandatory diagnostic accuracy metrics.** The study provides comparative data on biomarker levels but lacks a 2x2 contingency table or ROC analysis [Savrun 2017].
Frunza et al. (2024)	Database Search	**Lacks mandatory diagnostic accuracy metrics for AKI. The study provides comparative data on biomarker levels and general ROC curves for perinatal pathology severity, but lacks a 2x2 contingency table or specific ROC analysis for KDIGO-defined AKI vs. non-AKI**
Stryzhak et al. (2022)	Database Search	**Lacks mandatory diagnostic accuracy metrics. The study provides comparative descriptive data (medians and p-values) on biomarker levels over time, but lacks a 2x2 contingency table or ROC analysis evaluating predictive or concurrent accuracy for KDIGO-defined AKI**
Xu et al.2023	Database Search	**Different study design. The study proposes and evaluates a cystatin C-based AKI definition (CyNA: serum cystatin C >=2.2 mg/L or a >=25% increase) against in-hospital mortality outcomes, rather than testing serum cystatin C against a creatinine-based reference standard. It reports no sensitivity, specificity or AUC for serum cystatin C.**
Treiber et al. (2014)	Database Search	**Different study design. The receiver operating characteristic analysis reported in this study compares neonates with perinatal hypoxia/asphyxia against healthy controls, as stated in the paper and in its figure legend, rather than neonates who developed AKI against those who did not. The paper does not define, count or analyse AKI in its own cohort, so it provides no estimate of diagnostic accuracy against a creatinine-based reference standard.**

Table S1: List of Excluded Studies (Full-Text Assessment)
