# Supplementary material for: Serum Cystatin C as an Early Predictive Biomarker for Acute Kidney Injury (AKI) in Neonates: A PRISMA-DTA Systematic Review
Source: Children (Basel). 2026 Sep 5;13(9):1202. doi: 10.3390/children13091202 (PMC13605153; doi:10.3390/children13091202)
Supplement: Supplementary file 1 [file children-13-01202-s001.zip › Supplementary-Table-S2_2x2-data_2026-08-25.pdf]

Supplementary Material Table S2

Reconstructed 2x2 diagnostic data and study-specific accuracy for serum cystatin C

Study	TP	FP	TN	FN	Sensitivity (%)	Specificity (%)	AKI / non-AKI
Elmas et al., 2013	1	10	46	5	16	82	6 / 56
El-Frargy et al., 2015	17	2	8	3	85	80	20 / 10
Abdelaal et al., 2017	24	13	63	0	100	83.3	24 / 76
El-Gammacy et al., 2018	12	2	60	1	92.3	96	13 / 62
El-Sadek et al., 2020	16	0	60	14	53.3	100	30 / 60
Nour et al., 2020	12	4	12	2	86	75	14 / 16
Zhang et al., 2020	23	32	71	14	61.5	69.3	37 / 103
Diab et al., 2022	18	0	72	0	100	100	18 / 72
Refat et al., 2023	20	3	46	1	95	94	21 / 49
Wang et al., 2020	23	13	22	4	85.2	62.9	27 / 35
Hidayati et al., 2021	27	39	64	5	84.8	61.8	32 / 103
Abdelsattar et al., 2025	46	12	36	6	88.46	75.0	52 / 48

TP, true positives; FP, false positives; TN, true negatives; FN, false negatives.

The 2x2 cells were reconstructed from each study's published sensitivity, specificity and group sizes; none of the included studies publishes a 2x2 table directly. The final column gives the denominators used, which are the comparison groups on which each study computed its receiver operating characteristic analysis rather than total enrolment. For Wang et al. this is the 27 neonates with AKI against the 35 without, within the severe hyperbilirubinemia subgroup; for Abdelsattar et al. it is the 52 septic neonates with AKI against the 48 without. Sarafidis et al. is not tabulated: that study publishes no 2x2, its AKI group of eight cannot yield the published sensitivity of 66.7%, and its serum cystatin C receiver operating characteristic did not reach statistical significance (AUC 0.731, p = 0.067). Each reconstructed pair was checked against the accuracy figures published by the source study: across the twelve rows the sensitivity and specificity recomputed from the integer cells differ from the published values by at most 0.8 percentage points, which is the rounding error expected when integer counts are fitted to a published percentage. Sarafidis et al. is deliberately absent: that study publishes no 2x2 table and a group of 8 neonates with AKI cannot produce a sensitivity of 66.7% on integer counts.
