# Supplementary material for: Serum Cystatin C as an Early Predictive Biomarker for Acute Kidney Injury (AKI) in Neonates: A PRISMA-DTA Systematic Review
Source: Children (Basel). 2026 Sep 5;13(9):1202. doi: 10.3390/children13091202 (PMC13605153; doi:10.3390/children13091202)
Supplement: Supplementary file 1 [file children-13-01202-s001.zip › Supplementary-Table-S3_Funding_2026-08-25.pdf]

Supplementary Table S3. Funding sources reported by the included studies

Study	Funding source/status reported in the primary article
Abdelaal et al., 2017 [16]	Not reported
El-Gammacy et al., 2018 [10]	Not reported
El-Sadek et al., 2020 [17]	None declared
Elmas et al., 2013 [18]	Not reported
Nour et al., 2020 [19]	No funding was secured for the study
Sarafidis et al., 2012 [20]	Not reported
El-Frargy et al., 2015 [21]	Not reported
Refat et al., 2023 [2]	Not reported
Hidayati et al., 2021 [22]	Not reported
Diab et al., 2022 [24]	No funding
Wang et al., 2020 [25]	Not reported
Zhang et al., 2020 [26]	No funding was received for the study
Abdelsattar et al., 2025 [15]	No external funding

Note: "Not reported" means that no explicit funding source or funding statement was identified in the published article; it should not be interpreted as evidence that the study received no funding.
